# Using a Clinical Formulation to Understand Psychological Distress in People Affected by Huntington’s Disease: A Descriptive, Evidence-Based Model

**DOI:** 10.3390/jpm12081222

**Published:** 2022-07-27

**Authors:** Maria Dale, Ashleigh Wood, Nicolò Zarotti, Fiona Eccles, Sarah Gunn, Reza Kiani, Amanda Mobley, Noelle Robertson, Jane Simpson

**Affiliations:** 1 Huntington’s Disease Service, Leicestershire Partnership NHS Trust, Mill Lodge, The Rise, Narborough, Leicester LE19 4SL, UK; ashleigh.wood6@nhs.net (A.W.); sarah.gunn@leicester.ac.uk (S.G.); reza.kiani@nhs.net (R.K.); amanda.mobley@nhs.net (A.M.); 2 Department of Neuroscience, Psychology and Behaviour, University of Leicester, University Road, Leicester LE1 7RH, UK; nr6@leicester.ac.uk; 3 Division of Health Research, Faculty of Health and Medicine, Lancaster University, Lancaster LA1 4YG, UK; nicolozarotti@gmail.com (N.Z.); f.eccles@lancaster.ac.uk (F.E.); j.simpson2@lancaster.ac.uk (J.S.)

**Keywords:** Huntington’s disease, formulation, challenging behaviour, biopsychosocial, psychological distress

## Abstract

Huntington’s disease (HD) is an inherited, life-limiting neurodegenerative condition. People with HD experience changes in cognitive, motor and emotional functioning, and can also, mainly at later stages, exhibit behaviours that professionals and carers might find distressing such as hitting others, throwing objects, swearing or making inappropriate comments. While clinical formulation (an individualised approach used by mental health professionals to describe an individual’s difficulties) is a helpful tool to conceptualise patients’ wellbeing, a specific formulation framework has not yet been developed for HD. However, evidence has shown that formulation can help guide clinical interventions and increase consistency of approach across multi-disciplinary teams, refine risk management, and improve staff or carers’ empathic skills and understanding of complex presentations. As a consequence, this paper proposes a new clinical formulation model for understanding distress among people with HD, based on a biopsychosocial framework. More specifically, this includes key elements centring on an individual’s past experience and personal narratives, as well as anticipatory cognitions and emotions about the future. In-depth discussions regarding the components of the model and their importance in HD formulations are included, and a fictional yet representative case example is presented to illustrate their application within the context of personalised care.

## 1. Introduction

Huntington’s disease (HD) is a complex genetic neurodegenerative disease which is transmitted via an autosomal-dominant mechanism. It causes a wide range of motor, cognitive, and psychological difficulties which, over time, lead affected individuals to require 24-hour care and support [1]. Physical symptoms (required for clinical diagnosis) typically appear around age 30–50 (although “juvenile” and late onsets have also been described) [2,3] and include poorer movement control, chorea (involuntary movements), bradykinesia, continence issues and dysarthria [4,5]. Following this, the average life expectancy of people with HD (pwHD) is approximately 15–20 years [1]. No cure is currently available for HD, although a number of promising disease-modifying treatments are in development [6]. 

From the age of 18, those who are at risk of HD can undertake predictive genetic testing to determine whether they have the HD gene expansion. For the purposes of this article, we will refer to people who have the gene expansion but not the clinical diagnosis as “premanifest HD”, and those with the clinical diagnosis as “manifest HD”. 

### 1.1. Psychological Distress among pwHD

Evidence has shown that the cognitive, emotional, and behavioural difficulties experienced by pwHD may precede the onset of motor difficulties by many years [4,7,8,9]. Difficulties associated with HD can include emotional lability, irritability, anxious or obsessive-compulsive thoughts, low mood, demotivation and apathy and, less commonly, psychosis [10,11,12,13]. These can be highly challenging for both pwHD and those providing care and support [14,15,16,17]. Many of these difficulties may be associated with a wide range of losses linked with HD, which not only affect individuals directly (e.g., loss of family members to HD and legacy fears), but also crucial components of psychosocial functioning such as independence, communication, identity, and roles [18]. 

Other distressing behaviours (the term “challenging behaviours”, although arguably more common, has not been adopted in this article due to its blaming connotations) may be observed as the disease progresses, which translate into significant difficulties for families or carers to manage. These may include aggression, screaming, repetitive actions or phrases, destructive or dangerous behaviours, removing clothes in public or other disinhibited behaviours [19]. While these are traditionally viewed as a function of neurological changes in pwHD, more psychologically informed frameworks have begun to conceptualise how psychological distress, unmet needs and social context may coalesce with cognitive and organic changes to lead to expressions of distress through behaviour [20,21]. 

However, due to the complexity of HD and its associated difficulties, as well as its chronic trajectory, developing appropriate and meaningful clinical understandings of presentations remains challenging. 

### 1.2. Clinical Formulations

Clinical formulations are an approach used by mental health professionals to conceptualise psychological difficulties [22]. They comprise the development of hypotheses on the nature and causes of a person’s clinical presentation based on relevant theories as well as an in-depth clinical assessment, exploring the role of past experiences, triggers for distress, maintaining factors and potential targets for intervention [23]. Formulations are used as an alternative or addition to psychiatric diagnoses, and are useful for directing psychological interventions as well as other approaches across the multidisciplinary team [24]. A clinical formulation is often developed collaboratively with a patient, carer, or team [23,24] and is considered an iterative process rather than a finalised document, being dynamic and open to revision (“reformulation”) throughout the assessment or intervention [22]. In terms of theory, within the field of chronic illness, many approaches to formulation have been developed and these are often based on the biopsychosocial model. 

### 1.3. The Biopsychosocial Model

The biopsychosocial model was introduced as a progression from the biomedical model, and stresses the interconnectedness of biological, psychological and socio- environmental factors to help explain health difficulties [25]. As a model of understanding psychological distress, it has a long history in the field of mental health [26] and has been applied across other neurodegenerative conditions that, like HD, affect cognition, movement and emotion. This includes dementia populations [27,28], Parkinson’s disease [29] and multiple sclerosis [30]. Due to the varying contributions of biological, psychological, social and environmental factors to the distress experienced by pwHD, the biopsychosocial approach has been recognised as a helpful guiding principle to inform HD care and formulation [31].

HD shares commonalities with these other neurodegenerative diseases, such as a progressive decline in abilities, no current cure and common anxieties and uncertainty about the disease course [32]. However, there are also important differences. Compared to other diseases, HD has an especially high burden on familial carers [33]. Moreover, the inherited nature of HD has implications for potentially multiple generations of family members being affected [34,35]. Since any child of an HD parent has a 50% chance of inheriting the gene expansion themselves, this situation commonly results in pwHD having previously witnessed a family member in advanced stages, potentially conferring deleterious effects on pwHD in terms of anticipatory grief and anxiety regarding the future and their disease trajectory [34]. These disease-specific factors warrant special consideration within the formulation of distress among pwHD. However, while some authors have previously highlighted the potential benefit of biopsychosocial approaches to understanding HD [36,37], a specific conceptual framework or formulation model has not previously been articulated.

### 1.4. An HD-Specific Formulation Model

The overarching aim of this article is to provide the first HD-specific clinical formulation model, using a multifactorial approach, to understand psychological distress among pwHD. This model was developed from clinical work undertaken by authors (MD, AW, RK) based in a specialist UK HD inpatient unit. While the model originally aimed to capture the various factors that could contribute to distressing behaviours among people with manifest HD, we believe the framework has wider applicability. It is, therefore, proposed that the model be used flexibly to help formulate a variety of psychological issues experienced among pwHD, across various stages of the disease, including those with premanifest HD. 

Each component of the model is described and situated within the HD literature, and the wider research into factors contributing to emotional distress and distressing behaviours is discussed. Due to its multifactorial nature, the model presented below lends itself particularly well to multidisciplinary or team formulation approaches, which become increasingly important as HD progresses and loss of insight into symptoms develops. Nevertheless, the model can also be used collaboratively in one-to-one sessions with a clinician (usually a psychological practitioner) and pwHD, and/or with carers and family members. Different applications and contexts for the formulation model are outlined further in the discussion.

## 2. Assessment 

A formulation is only as robust as the information on which it is based. Accordingly, completion of a meaningful, high-quality formulation is reliant on a thorough and holistic assessment derived from multiple sources. If the formulation is being used within the context of multidisciplinary teamwork, then different professionals should contribute their knowledge and observations to ensure results from specialist cross-disciplinary assessments are included [38]. Sources of assessment information for inclusion may comprise clinical interviews, behavioural observations, physical assessments, risk assessments, psychometric tools, neuropsychological or cognitive assessment, objective medical examinations, clinical notes, information gathered from carers and relatives, feedback from diaries or homework tasks and any other potential assessment sources (if available) such as remote sensor monitoring (e.g., digital wristbands and other wearable devices). This produces the most complete picture possible of the individual’s current physical, cognitive, emotional and behavioural wellbeing, to underpin the generation of hypotheses about the various contributions to the person’s psychological presentation. It is also crucial within the assessment process to gain an appreciation of positive or “protective” factors, i.e., an individual’s strengths and resources, to help understand the person as a whole and in context. This also helps circumvent arguably one-dimensional “problem-focused” approaches, that centre on the disease rather than creating a dynamic formulation of the individual and the factors influencing their wellbeing. 

## 3. Presenting the Model 

### 3.1. Model Overview

**Figure 1:**
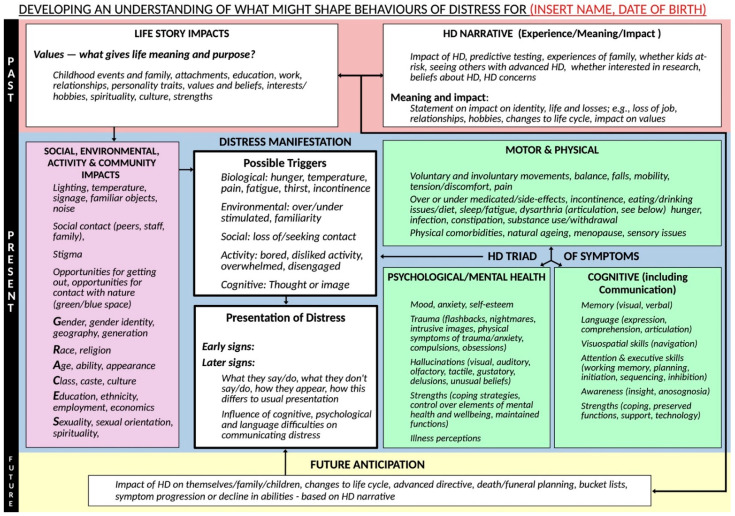
Template formulation model (prompts for completion in italics).

First, a brief overview of the framework will be presented, before discussing the individual components in more depth. A blank template is provided in Figure 1 with an extensive list of considerations for each element of the model, providing prompts and guidance for completion (in italics). 

In the discussion section we also detail a fictitious, but representative, case example to demonstrate how the model can be used. 

The model is divided into past, present and future (indicated to the left of the diagram)—an approach supported by prior research showing that the passing of time is central to adjustment among people who live with HD [39]. These temporal components seek to recognise the impact of past experiences and future anticipations on pwHD, and how these might guide us to understand a particular presentation or behaviour occurring in the present. While past and present experiences are usual features of clinical formulations, our model proposes that future-oriented cognitions and emotions are also crucial in understanding the experience of pwHD [39,40], given the nature of the neurodegenerative process and also the potential of future generations being affected. 

For each component of the model, the pwHD’s abilities, strengths, and awareness of difficulties should be considered with respect to co-development of coping strategies and compensatory tactics. For example, an individual may have dysarthria and reduced language abilities but learn to utilise communication aids. Similarly, a person may experience increased involuntary movements when anxious, which can be partially mitigated by using relaxation exercises to reduce worry and associated physical symptoms. Importantly, insight may vary across symptoms; an individual might be aware of their balance or coordination difficulties, but have limited awareness into cognitive, behavioural or emotional changes; anosognosia can also be highly granular, specific to individual difficulties within these domains. Moreover, for some pwHD there might be discrepancies between their metacognitive awareness (i.e., knowledge—they have learnt and can consequently report the sorts of difficulties they have) and their online awareness (i.e., ability to recognise they are having a difficulty at the time they are having it) [41]. 

The information contained in the formulation is presented in a dynamic and integrated manner, demonstrating relationships between different factors rather than listing difficulties and strengths [22]. Importantly, while we have found the diagrammatic model a useful tool to support clinical discussions, it is also appreciated that completing a set of boxes to describe complex experiences and difficulties might appear reductionist. This model can, therefore, be used to guide more narrative formulation approaches alongside further nuanced discussion, such as within psychological or psychiatric reports.

### 3.2. Conceptualisation of Present Difficulties

The “Present” components of the model aim to reflect an individual’s current experiences, taking into account both their internal and external world in the here-and-now. An individual’s distress presentation summarises the salient psychological issues. This could be behaviour that is experienced as challenging by others (e.g., care staff or family members), but it could also be internal experiences self-reported by the pwHD. Distress and possible unmet needs are considered in light of the pwHD’s current HD symptoms, environment, social contact, opportunity for activity and community understandings. 

#### 3.2.1. Distress Presentation

With respect to other psychological treatment models that address distressing behaviours (e.g., positive behaviour support), our formulation template requires a description of how distress presents in the individual currently (“Presentation of distress”). This is generally a helping starting point for formulation and a stimulus for discussions with pwHD, HD family members, or a multidisciplinary team. In some cases, distressing behaviours might not appear to cause distress to the individual themselves, but others might find them distressing (e.g., repetitive or aggressive behaviours). Differences in personality, cognition and communication also provide important evidence of changes in emotional wellbeing for the pwHD and should be attended to carefully, bearing in mind that pwHD often underestimate or have limited awareness/insight into their symptom as their condition progresses. A family member or familiar carer may be helpful in providing further information [42,43]. Importantly, while distressing behaviours may reflect the mental health difficulties associated with HD, this is not assumed, and both “Presentation of distress” and “Psychological/mental health” are accommodated in the diagram (the latter is discussed in more detail below). This is because distress might be influenced by a range of factors unrelated to the “neuropsychiatric” component of the HD triad of symptoms, such as difficulties in communication, lack of control, grief and loss (whether current or anticipatory) or restrictions. 

Feeding into the box describing “Presentation of distress” is the “Triggers” section. Information from other components of the formulation model that have been identified as precipitating distress will feed into the “Triggers” section. “Triggers” information can be heavily supported by input from pwHD, family members, and carers if appropriate. Behavioural monitoring charts (usually completed by staff or carers) with an “Antecedent, Behaviour, Consequence” approach can also help identify potential short-term and long-term triggers. Examples are provided in the template, but include considerations relating to biological triggers (e.g., hunger, thirst, temperature, pain and discomfort, incontinence), environmental changes (e.g., noise levels, unfamiliarity with surroundings or people), issues around social contact, and activity levels (e.g., boredom and under-stimulation, or overstimulation and disengagement). 

#### 3.2.2. HD Symptoms

Symptoms of manifest HD are often classified into three groups: physical, cognitive, and psychological [44,45]. All three can have significant impact on the frequency, severity and form of distress. Our model considers the three components as separate but integrated, acknowledging the interrelated complexity of the three elements of HD-related wellbeing. 

##### Motor and Physical Symptoms

Motor symptoms include changes in balance and mobility, the presence, frequency and intensity of involuntary movements (“chorea”) and the muscle groups affected, and changes in continence, communication, fine motor movement, eating and drinking. A rounded assessment of physical health should also consider supplementary physical factors, such as changes in energy, pain or discomfort and sensory issues. The effects of comorbid health diagnoses and medication (for HD or other conditions) should also be considered; for example, some medications can cause lethargy, which might be mistaken for an increase in apathy, low mood, or difficulties with movement.

Additional considerations include the role of diet and nutrition [46]. For example, hunger and thirst can trigger distress, and low weight is correlated with chorea [46,47]. Higher body mass index (BMI) has been shown to predict higher levels of irritability and apathy in people with manifest HD (and also apathy in premanifest pwHD) [42]. Diet quality can also affect mental health, and numerous pathways may mediate this relationship [48], with potential relevance for pwHD [49].

##### Cognitive Symptoms

HD is associated with a wide range of cognitive impairments, which progress over time. These may be observed especially in the domains of memory, psychomotor speed, executive functioning and, in later stages, language [50]. While premanifest individuals usually do not report significant issues with language or long-term memory, early impairments involving working memory and executive functioning have been described [51,52].

In addition, people with manifest HD often show early significant difficulties in recognising emotions [53], especially negative ones such as fear, disgust and anger [54,55]. This is particularly evident in studies examining interpretation of facial cues [56], but also in other modalities including auditory stimuli [57] and body language [58]. Similar studies involving people with premanifest HD have shown less consistent findings, with some reporting selective impairments for negative emotions and disgust in particular [59,60] and others showing no significant issues [61]. In addition, difficulties involving theory of mind (i.e., predicting and understanding other people’s mental states, intentions, and reactions) as well as pwHD’s awareness of their own impairments (“anosognosia”) may be observed [19,62]. 

Changes to cognition substantially affect how pwHD interpret and respond to their environment. For example, an individual with executive functioning difficulties who is unable to initiate tasks may not be able to use a call bell to request assistance with toileting or mobilising. Similarly, those with problem-solving difficulties may not be able to respond appropriately to discomfort, such as removing or adding clothing due to uncomfortable temperatures. These cognitive difficulties may influence an individual’s independence in activities of daily living if they require prompting or assistance, reduce an ability to work or partake in leisure activities and interrupt relationships or social interactions. All of these can lead to distressing behaviours, generated through grief or frustration at losses or restrictions. There are also implications for risk management, where pwHD may not be able to judge risk accurately or may undertake actions impulsively as they become more disinhibited over time. Finally, in the later stages of HD when disorientation becomes more problematic, resultant confusion and anxiety can be a significant trigger for distress-related behaviours.

##### Communication Difficulties

The heterogeneous difficulties experienced by pwHD impact multiple aspects of interpersonal communication including language production, speech articulation and the aforementioned recognition of verbal and nonverbal emotional cues [57,63]. Despite this, current evidence regarding communication for pwHD appears largely focused on objective assessments of impairments from a clinician’s perspective, rather than the subjective experience of affected individuals and the people around them [64,65]. The limited studies that have focused on the latter reported that communication in pwHD was negatively influenced by various psychosocial factors including isolation, lack of discourse initiative and support, fatigue, depression, personality changes, emotion dysregulation and loss of perceived control [18,66,67]. In addition, these factors have shown potential to exacerbate family issues when effective communication is required while building narratives around the disease and genetic risk within the family system, especially with children or adolescents [68].

Loss of the ability to communicate has clear implications for being unable to express thoughts or emotions, or to get needs met quickly, easily, or at all. PwHD may struggle to find the right words for what they wish to say, may produce them in the wrong order, or may be unable to express the words with the necessary clarity for others to understand. Individuals may also increasingly struggle when provided with too much information, or too quickly, as well as struggling with comprehension of more complex linguistic constructions such as abstract concepts, negatives (“not”, “don’t”) or references to time (“yesterday”, “this afternoon”). Social constructs such as sarcasm or irony can also be highly challenging, as they require interpretation of potentially complex expression and context. These difficulties can lead to tension, misunderstandings and distress for the pwHD and those close to them, with a clear route to distressing behaviours relating to frustration, discomfort and unhappiness. Some evidence has shown positive results for the use of augmentative and alternative communication (AAC) to facilitate communication between pwHD and their carers [69,70]. 

##### Psychological and Mental Health

The “Psychological/mental health” aspect of the formulation model has a close relationship with the “Presentation of distress” section, but as described earlier, they can differ. 

A wide range of mental health difficulties are associated with HD, including anxiety, depression, apathy, irritability, obsessive-compulsive and perseverative behaviours, and (less commonly) psychosis. A large European study (REGISTRY) found 87% of gene expansion carriers had some form of reported psychological difficulty [71] and such difficulties are key predictors of quality of life in pwHD [72,73]. Importantly, with the exception of apathy [12,74], the vast majority of research has demonstrated that mental health symptoms are not directly linked with HD progression (unlike physical, cognitive and functional changes) [75,76,77]. The cause of these difficulties is likely to be multifactorial, due to structural brain changes, cognitive and sensory changes and the extremely difficult experience of living with the condition [75]. 

Depression and suicidal ideation: Depression and low mood are very common in pwHD [1,12], but prevalence estimates are highly variable depending on the methods, definitions and populations studied. For example, one cross-sectional study found moderate to severe depression in 13% of manifest individuals, while a longitudinal study of individuals with depressive symptoms found rates of 60% over time [76]. In addition, depression can be difficult to identify clinically as behavioural indicators may overlap with other HD-related difficulties, such as insomnia, slowed movements and low motivation [78].

While neurological changes certainly contribute to low mood, higher rates are reported around the time of onset, which probably also reflect individuals struggling to come to terms with being “diagnosed” with HD, as well as the beginning of cognitive difficulties and increased consequent work and home stresses at this time [75,77]. 

Risk of suicide and suicidal ideation is also high compared both to the general population and other neurodegenerative conditions, with 20–30% of manifest individuals reporting suicidal ideation and high rates throughout the disease course [79]. Studies vary in opinion regarding whether the risk varies with stage of illness, with some suggesting the onset of symptoms and subsequent loss of independence could be key triggers for an increase in suicide and suicidal ideation [80]. However, some studies find no difference in rates by stage of illness [79]. Discussions around assisted dying and death are often welcomed by pwHD [81] and would be addressed in the “Future anticipations” element of the formulation. 

Anxiety and worry: Anxiety is also common in HD, though less well studied than depression. Again, prevalence reports vary hugely from 13–71% in manifest pwHD [82]. Generalised anxiety disorder and panic disorder in particular are frequently experienced [82]. Anxiety does not seem to be consistently related to cognitive function and there is no clear change in prevalence across the disease course [82]. Many worries of individuals are understandable, such as concerns about genetic discrimination, financial worries, concerns regarding future loss of function and impact on family members. In fact, many of these concerns are understandably shared within members of HD families (those with and without the gene expansion) [33,34,39,83,84]. 

Irritability and aggression: The concept of irritability has not always been well defined in HD and is related to constructs such as “anger” and “frustration” [21]. Irritability towards the self and others, and associated aggressive outbursts, can be some of the most troubling for family members to cope with and can contribute to family discord, work stress and relationship breakdown [75]. Prevalence statistics vary [85]; with up to 83% of people with manifest HD at any timepoint across longitudinal assessments [76]. In the early disease stages, irritability may be in part a response to struggling with cognitive or physical changes, or indeed be associated with low mood or depression [21]. It is important to note that pwHD tend to underestimate any symptoms of irritability in themselves, which can be corrected upwards by an informant if present, but limited awareness into this domain may feed into the inconsistent findings around prevalence of irritability [42].

Aggression (an overt behaviour, as opposed to irritability, a mood state) is also experienced by some pwHD. A systematic review suggested rates range from 23% to 65% across studies [86]. Both verbal and physical aggression occur, with verbal aggression perhaps having higher prevalence [86]. Studies report mixed findings as to whether aggression is most common in early, mid or later stages [86], which again raises the likelihood that aggressive behaviour is rooted in multifactorial causes rather than being a direct symptom of disease progression. When completing the formulation, as aggression is an overt and risky behaviour, if present, it can often be the salient psychological issue. In these instances, aggressive behaviours would be detailed under the “Presentation of distress” section. 

Apathy: Apathy, defined as a lack of motivation leading to reduced goal-directed behaviour, cognition and emotion [87], occurs across the disease course. It can manifest as reduced activity, social withdrawal, a loss of interest in previously enjoyed activities and reduced attention to self-care. Some studies find it is the most common difficulty [12] and it is the one psychological difficulty clearly associated with disease progression [12,75,76,88]. Some of the symptoms may overlap with depression, but depression and apathy do not correlate, follow different typical patterns over the disease course and emerge as different factors in analyses [10,76,88].

Perseverative and/or obsessive-compulsive behaviours: HD-related cognitive difficulties can include impaired executive function. As a result, pwHD can exhibit perseveration and problems with set-shifting or changing task [75]. Consequently, people with HD can appear “obsessed” with certain actions (e.g., repeatedly asking about something or appearing fixated on a plan or schedule), which can cause great difficulties for families and carers [89]. These behaviours often lack features of obsessive-compulsive disorder, i.e., pwHD do not always see the behaviours as abnormal, or try to resist them, but they are often reported or misunderstood as obsessive-compulsive symptoms [76]. A recent review calls for a greater distinction between the obsessive-compulsive and perseverative behaviours on this basis [90]. 

Prevalence rates for obsessive-compulsive behaviours range from 5–52% and up to 70% for perseverative behaviours [90], so these represent a significant consideration when formulating distressing behaviours in pwHD. They tend to increase with more advanced HD (although may decline in the very late stages of the disease).

Psychosis: Hallucinations and delusions can occur as part of HD, though the occurrence appears rare than for other difficulties, with prevalence figures of 3–11% [91]. Some studies show psychosis is associated with mid-stages of HD [12], but others that it predates motor symptoms [91]. For a small group of families, it appears to be the predominant symptom and may be the first symptom to appear across generations [92].

When assessing clinically, it is important, however, to consider other provenances of symptoms relating to “psychosis”, such as a history of trauma (e.g., physical or sexual assault). 

#### 3.2.3. Social, Environmental, Activity and Community

This section of the formulation model encapsulates the person’s social circumstances and contacts, environments in which they are residing or spending time (and sources of discomfort or distress), activities (or restrictions on activities) and the impact of the wider community. A crucial factor embodied within this facet of the model is the potential impact of the people with whom the person has contact and how they approach them, deal with HD or, in the case of paid carers or hospital staff, how their approach is tailored to the person’s care needs. These factors interrelate dynamically with other facets of the model, notably how much or little the person’s current social circumstances reflect their life story, interests and values, how well they are able to address difficulties independently or recruit support to do so, and how their current situation and symptoms interact with their narratives about HD. 

We also consider any impacts of a person’s “Social GGRRAAACCEEESSS”, comprising their gender, geographical situation, race, religion, age, ability, appearance, class, culture, ethnicity, education, employment, sexuality and sexual orientation and spirituality [93]. These characteristics impact on a person’s self-perception, and on the stigma and discrimination which may be received from others around them—including potential genetic discrimination, which might generate shame or embarrassment [94]. As a genetic disorder, HD may differentially affect people based on factors such as sex, income and race/ethnicity [95].

Further, the relationship between deprivation and distress is complex and difficult to disentangle in terms of cause and effect [96]. Deprivation relates not only to wealth and housing but can also include exposure to stressors such as violence, crime or lack of public green space. HD has implications for mental health and disability, which both have a complex interaction with deprivation factors; for example, it is known that experiencing disadvantage increases the risk of mental health problems [97]. Poor mental health and high levels of psychological distress can lead to a “spiral of adversity” where factors such as employment, housing, income and relationships are affected by their condition, which then further negatively affect mental wellbeing. All such social factors have relevance to HD, as the motor symptoms usually start to affect employment, housing, income and relationships in mid-adulthood. Accordingly, it is important to consider such social impacts when developing a multifactorial formulation of distress among pwHD. 

The social model of disability has additionally been proposed as having relevance to the psychological needs of pwHD [98]. This model does not just argue that social factors affect the mental health of people with impairments, but that society’s stigmatising attitudes towards individuals with any physical difference actively disables individuals. People are therefore not intrinsically “disabled” but become disabled by society. 

A further consideration within this section of the model is access to nature. The benefits of nature contact to mental wellbeing have been well-documented [99,100], with the level of nature connectedness having particular significance [101]. Access to nature may be limited for pwHD due to physical disability and/or due to residing in care facilities where trips out may be limited by staff resourcing difficulties. This factor should be included in a formulation relating to distress for pwHD, especially if connection with nature is consistent with the person’s values and life history.

### 3.3. Role of the Past in Conceptualisation of Present Difficulties

Past elements of the formulation are indicated in the upper section of the diagram (Figure 1), and seek to define an individual’s prior experiences and narratives which are likely to influence their understanding and interpretation of themselves, others and the world. 

These experiences, narratives and values relate to both HD and an individual’s holistic life-story. In the model, we have explicitly parsed out “HD narratives” from the more generic “Life story”. Although we acknowledge these are highly interlinked, we believe that special consideration of the specific impacts of HD is important to emphasise, particularly if using this diagram to co-formulate pwHD within a staff group where distressing behaviours might be occurring. This specific focus on HD-related distress helps staff and pwHD consider the influence of HD on the wider family context, and how HD can overshadow decisions and approaches to life even well before genetic testing takes place. Moreover, this section can help promote a deeper understanding of how HD might have shaped a person’s life long before any psychological difficulties, which can be especially helpful in the context of supporting engagement and empathy in hospital and care home staff.

#### 3.3.1. Life Story

This section presents important components of an individual’s history and personality. Past life experiences influence our identity and help us understand who we are. Understanding a person’s life history becomes even more important in people with conditions such as HD, where over time there is cognitive decline and communication becomes more limited. Although under-researched among pwHD, in other motor neurodegenerative conditions, such as Parkinson’s disease, retaining a sense of self is important in the adjustment process [102]. Maintaining a sense of cohesion regarding interests, background, previous roles, strengths, achievements and values can all contribute to psychological health, especially when people experience memory difficulties and/or are unable to communicate important aspects of their identity and values. This can be supported through life story work with the individual, family and staff members to build a personal biography and a timeline of key past life events.

Broadly, the formulation should be constructed from a positive position, emphasising the individual’s strengths, successes and sources of support. However, difficult experiences—particularly those that may have been traumatic—should also be included where they may offer opportunities to understand an individual’s beliefs, feelings and actions. This is important when considering how such events may shape distressing behaviour and responses to particular stimuli. Pertinent experiences from childhood and adulthood may be included, as well as consideration of family, friends, attachments and overall experiences of others. For example, the role of attachments and childhood experiences on adult mental wellbeing among pwHD has been demonstrated [103], although again this area is presently under-researched in HD populations.

A number of psychological approaches that have been considered useful for pwHD and other neurodegenerative conditions take a values-based approach, and include acceptance and commitment therapy (ACT), narrative therapy (NT), compassion-focused therapy (CFT) and mindfulness-based approaches [104,105,106,107]. These focus on supporting individuals to explore their history, values, beliefs and mantras, and how these impact on the individual’s principles, interests, hobbies and strengths. This crucial information should be included and highlighted as part of the formulation, again helping bring pwHD into focus for those supporting them and helping to guide interventional approaches.

#### 3.3.2. HD Narrative, Meaning and Impacts

This section of the model includes people’s experience of HD in their family, including learning about HD and personal risk, undergoing genetic testing, and knowledge of other family members’ experiences of HD. It examines the meanings that people have consequently attached to the condition, and narratives they have developed around it. This section also encourages the completion of a statement about the impact that HD has had on the person, such as losses of roles or functioning. 

Several studies have investigated the role of factors relating to identity among pwHD, the narrative they hold around their condition and how this can relate to distress [36,108,109]. Several key concepts from health psychology have been applied, evidencing predicted relationships between these narratives and psychological distress seen in other neurodegenerative conditions. For example, using the Self-Regulatory Model [110] as a theoretical framework, several studies have revealed that pwHD perceived a high number of symptoms to be associated with the condition, reported a high level of perceived consequences and expressed belief in long disease duration [36,108,109]. In addition, Arran and colleagues [36] found that pwHD reported little personal or treatment control of their illness, felt unable to make sense of their illness and had a stronger belief that their illness was chronic. Interestingly, although individuals recognised the cause of their illness to be hereditary, both Arran et al. (2013) [36] and Kaptein et al. (2006) [109] found some ambivalence regarding the endorsement of HD as a genetic condition. While a genetic cause is not in doubt, explanation may be found in a number of experiences, such as differences in age of onset even within the same family, adding complexity to individuals’ narratives around the condition’s onset.

Alongside these quantitative findings using standardised scales, qualitative studies have offered more complex and nuanced understandings. For example, Maxted et al. (2014) [94] described how families where the HD gene is present describe the condition as “a spectre hanging over us” (p. 342), with the condition causing the family to feel isolated with its own rules and practices and “us against the world” (p. 343). Ekkel et al. (2021) [40] also highlighted several strategies adopted relating to future identity. These involved keeping the knowledge of their future care needs at a psychological distance, so that life in the “here and now” remained manageable. 

Accordingly, the previously noted findings have relevance for clinical understandings and psychological interventions. For many pwHD, information about the disease and experiences of HD are likely to have arisen in the context of another family member, most frequently a parent or grandparent. An individual’s experience and education about HD, both in reference to themselves and others, is likely to affect the meaning drawn from the disease, and ultimately mediate the resulting impact on their mental wellbeing. This HD narrative is suggested to have a strong influence on both emotional difficulties, particularly acceptance of the diagnosis and disorder, and anticipatory emotions and cognitions regarding the future.

As noted above, illness identity can have a significant impact on emotional wellbeing. For example, individuals who experience HD as a hopeless, life-limiting, disabling illness are likely to form a strong negative narrative which may focus on loss of function and opportunities. These individuals are likely to struggle more with recognising their existing strengths and abilities. On the other hand, individuals who have experienced others with HD as capable, strong and able to find continued enjoyment in life, may be more likely to develop a positive narrative around the illness. While the roles of positive psychology constructs such as “optimism” “gratitude” and “hope” have only been explored to a limited extent within HD populations [84,111,112], these have been found to influence psychological wellbeing in other chronic conditions [113,114]. Again, an understanding of these perceptions and narratives about HD and its impacts can be crucial for the pwHD, their family members and their carers to understand its effects on distress in the present.

### 3.4. Role of the Future in Conceptualisation of Present Difficulties

The final strand of the model serves to emphasise that HD, as a life-limiting, degenerative condition, emphasises emotions and behaviours relating to future expectations and losses. PwHD are likely to hold powerful images and narratives about how their illness will develop in the future. These thoughts may reflect symptom progression, ability level and life expectancy, and are likely to be influenced by their HD narrative and experience of others’ illnesses. Similarly, pwHD may hold expectations about how HD will impact their family, children and friends, including fears for relatives at risk of the inherited condition. 

In working with pwHD, clinicians and other staff should again seek as appropriate to take a positive approach, thinking about goals that individuals hope to achieve, “bucket lists”, and otherwise planning for the future to get the most out of their time. In addition, positive planning in terms of advanced directives and funeral planning, ideally engaging family if this feels appropriate and welcome for all involved, to help promote a sense of control and agency for the pwHD. Again, a focus on skills and strengths which will be retained, alongside the inevitable losses, may help to promote wellbeing from the pwHD and empathy/engagement from staff working with them.

## 4. Discussion

Having outlined each component of the model, the various potential applications for the formulation framework will now be discussed. This is followed by a fictitious case example to help demonstrate how the formulation can be used. 

### 4.1. Model Applications, Implementation and Future Research

As mentioned above, the present model originated from a need for a formulation framework with specific relevance for the experience of people living with HD. As such, a number of potential applications can be identified for the model. One of these is to help supplement and structure team formulation sessions—i.e., a protected time for staff members to develop a collective understanding of the individual and determine an intervention plan. More specifically, team formulation sessions have been linked to numerous benefits for patient care [115], especially as they involve a range of different professions and potentially diverse theoretical perspectives, reducing the risk of important factors being missed or underestimated in their importance. Moreover, they can enhance team understanding of work with more complex patients, helping staff to consider risk-management more fully. Team formulations can also increase staff consistency of approach, challenge negative myths or assumptions about patients and enhance staff empathy and compassion. It should however be noted that formulation is an under-researched area and much evidence of these benefits to date is limited to clinical reports, audits and qualitative studies [115]. 

Given the limited research into the effectiveness of clinical formulations, it is recommended that further work is undertaken to evaluate this model. A range of methods could be used to help establish its effectiveness. Currently, the model has been implemented routinely in an inpatient unit in the UK and is being evaluated through initial feedback from clinical staff on their experiences of using the model, using both qualitative interviews and questionnaires. These will capture their views on whether the model has impacted on team understanding, consistency and other aspects of care. The model could be further evaluated through examining pre- and post- measures of multidisciplinary team or ward functioning, including the use of restrictive practices [116], in inpatient or residential care scenarios. Additionally, repeated measures that explore clinical outcomes for pwHD such as instances of distressing behaviour (e.g., aggression) could be used to ascertain effectiveness. 

A further application of the model consists of potential one-to-one collaborative work with the individual with HD (whether premanifest or manifest), if they are able to engage in the process. The prompts in the diagram can be used to form the basis of a clinical interview. The use of direct quotes from the individual can help provide collaborative person-centred care, where the person feels listened to and understood. Clinical work with pwHD often involves working with carers or family members. Where needed, the model can be used to help educate families about the range of factors that might contribute to a person’s distress. The tool can also be used collaboratively to summarise the challenges that families might be experiencing and to help provide empathy and understanding both for the person affected by HD and for the family members and carers. 

The voice of pwHD is essential in HD research [20] and it has been recommended that formulations should be explored with client focus groups to identify how they feel about clinical formulations [117]. Therefore, gaining a perspective from HD families on the usefulness of the model and whether they consider their care has improved as a result of the formulation would be beneficial. 

In terms of implementing this model, Aston (2009) [117], in a review exploring the efficacy of clinical formulations, identified how formulation guides might help improve the quality of formulations for both experienced and newly qualified practitioners. The model outlined in this paper might be particularly useful for clinicians less experienced with HD, and currently the authors of this paper are producing a formulation guide to help with implementation of the model. 

Aston (2009) [117] also recommended how the reliability and implementation of clinical formulation can be enhanced through training packages, using multiple perspectives to evaluate the formulation, videoing sessions and using clinical supervision. 

Although the model has a range of applications, it is acknowledged that there will be certain scenarios where the approach will have a low likelihood of being effective. This includes cases where the person cannot and/or is unable to engage in the process (e.g., due to limited communication skills), and where there is little known history and no connected persons to offer insight into their interests, background and experiences. In some instances, pwHD and their family members may find it difficult to engage with clinical services and therefore if clinical information is limited, then it will be difficult to implement this model successfully. 

### 4.2. Case Example 

**Figure 2:**
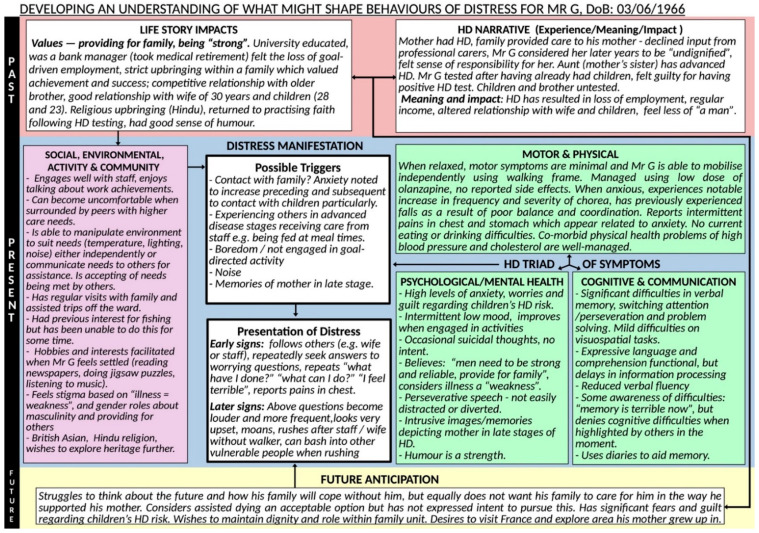
Case example of Mr G.

Here we present a fictitious case that is characteristic of the work that could be undertaken using this formulation model. Figure 2 illustrates the formulation template populated with the information from the case example.

#### 4.2.1. Background

“Mr G” is a 56-year-old British-born Asian gentleman, a bank manager by background, and father of two. He took early retirement as a result of HD symptoms and was admitted to a specialist HD hospital with concerns in relation to suicidal ideation, deterioration in his mobility, severe anxiety symptoms and family related feelings of guilt. He had inherited the HD gene expansion from his mother and witnessed the progression of the disease and her premature death, whilst adopting the role of her main carer.

#### 4.2.2. Factors Contributing to His Presentation

A multidisciplinary team formulation session using our proposed model was held two weeks after Mr G’s admission to inpatient care. The staff team comprised clinical psychologists, psychiatrists, nurses, speech and language therapists, physiotherapists, occupational therapists and activity co-ordinators. The model was used to develop a working hypothesis of potential factors adversely influencing his presentation. Mr G’s behaviours were discussed in the context of his life story and HD narratives, especially his beliefs around needing to be “strong” and providing for his family, and the relationship with adjustment to his HD symptoms and loss of roles. His negative illness perceptions of advanced HD as “undignified” (from experiences of caring for his mother), were linked to his fears about the future and the triggers for his distress (e.g., contact with advanced patients). The impact of his cognitive difficulties, particularly perseveration, were discussed in relation to his repetitive speech. Anxiety and guilt about his children’s risk were also considered important to his presentation. His strengths, particularly his humour, interests and spirituality were explored. The formulation session facilitated further discussion around developing a person-centred plan of intervention, incorporating various approaches suggested by different members of the team during the session.

#### 4.2.3. Intervention

As a result of this co-formulation session, a socio-cultural approach was added to the other models of care. This included environmental and structural adaptations (e.g., ensuring quiet times, adequate space from more vulnerable patients, routine), psychological therapies (e.g., strategies to cope with anxiety and guilt, mindfulness sessions), and pharmacological, psychiatric and medical interventions (e.g., medication for the management of his involuntary movements) to address Mr G’s complex presentation. Actions taken included discussion on maintaining his dignity based on his cultural and religious beliefs/heritage, facilitating regular family/community visits and providing opportunities to empower Mr G to recommence engaging in his interests and hobbies both on and off the ward.

## 5. Conclusions

The present article described the first formulation model developed specifically to help understand distress among individuals with HD. Using a temporal approach, the model encapsulates life story, HD-related experiences and narratives, socio-environmental factors, the triad of HD symptoms and future anticipations to understand HD-related distress. The resulting clinical tool can be used flexibly, according to specific clinical needs across a range of pwHD and service contexts, and can be used as an aide to the development of a narrative clinical formulation. Its successful implementation shows the potential to help enhance person-centred care for pwHD by increasing psychological understanding, enabling unmet needs to be identified, and offering avenues for intervention—all based on an individualised, evidence-based approach.

## Data Availability

Not applicable.
